# *N*-[4-Acetyl-5-isobutyl-5-(2-*p*-tolyl­prop­yl)-4,5-dihydro-1,3,4-thia­diazol-2-yl]acetamide ethyl acetate hemisolvate

**DOI:** 10.1107/S1600536808039998

**Published:** 2008-12-03

**Authors:** Mohamed Loughzail, Noureddine Mazoir, Celia M. Maya, Moha Berraho, Ahmed Benharref, Nouzha Bouhmaida

**Affiliations:** a Laboratoire de Chimie des Substances Naturelles, Faculté des Sciences Semlalia, BP 2390 Bd My Abdellah, 40000 Marrakech, Morocco; b Instituto de Química Física Rocasolano, Consejo Superior de Investigaciones Científicas, Serrano 119, 28002 Madrid, Spain; c Laboratoire de Chimie de Coordination, Unité Matériaux, Faculté des Sciences Semlalia, BP 2390 Bd My Abdellah, 40000 Marrakech, Morocco; d Laboratoire des Sciences des Matériaux, Département de Physique, Faculté des Sciences Semlalia, BP 2390 Bd My Abdellah, 40000 Marrakech, Morocco

## Abstract

The racemic  title compound, a new terpenoid, C_20_H_29_N_3_O_2_S·0.5C_4_H_8_O_2_, was synthesized from *Cedrus Atlantica* essential oil. The compound crystallizes with a disordered ethyl acetate solvent mol­ecule. The thia­diazole ring is almost planar, with a maximum deviation from the mean plane of 0.015 (2) Å for the C atom connected to the isobutyl group and has a puckering amplitude of 0.026 (2) Å. The dihedral angle between the benzene and thia­diazole rings is 18.32 (8)°. The crystal packing involves inter­molecular N—H⋯O hydrogen bonds.

## Related literature

For 1,3,4-thia­diazole derivatives and their biological activity, see: Abdou *et al.* (1991 ▶); Sakthivel *et al.* (2008 ▶); Tehranchian *et al.* (2005 ▶); Wang *et al.* (1999 ▶, 2004 ▶). For preparative methods, see: Beatriz *et al.*, 2002 ▶; Mohammed *et al.* (2008 ▶); For puckering parameters, see: Cremer & Pople (1975 ▶).
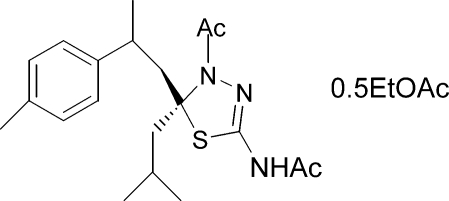


## Experimental

### 

#### Crystal data

C_20_H_29_N_3_O_2_S·0.5C_4_H_8_O_2_
*M* *_r_* = 419.57
Monoclinic, 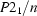

*a* = 7.8713 (3) Å
*b* = 12.7587 (5) Å
*c* = 22.9688 (9) Å
β = 90.937 (2)°
*V* = 2306.39 (16) Å^3^
*Z* = 4
Mo *K*α radiation
μ = 0.17 mm^−1^
*T* = 295 K
0.5 × 0.4 × 0.3 mm

#### Data collection

Bruker X8 APEX CCD area-detector diffractometer
Absorption correction: none
27541 measured reflections
7243 independent reflections
6688 reflections with *I* > 2σ(*I*)
*R*_int_ = 0.037

#### Refinement

*R*[*F* ^2^ > 2σ(*F* ^2^)] = 0.065
*wR*(*F* ^2^) = 0.143
*S* = 1.23
7243 reflections
298 parameters
H-atom parameters constrained
Δρ_max_ = 0.48 e Å^−3^
Δρ_min_ = −0.35 e Å^−3^

### 

Data collection: *APEX2* (Bruker, 2005 ▶); cell refinement: *SAINT* (Bruker, 2005 ▶); data reduction: *SAINT*; program(s) used to solve structure: *SHELXS97* (Sheldrick, 2008 ▶); program(s) used to refine structure: *SHELXL97* (Sheldrick, 2008 ▶); molecular graphics: *ORTEP-3 for Windows* (Farrugia,1997 ▶); software used to prepare material for publication: *WinGX* (Farrugia, 1999 ▶).

## Supplementary Material

Crystal structure: contains datablocks I, global. DOI: 10.1107/S1600536808039998/fj2168sup1.cif

Structure factors: contains datablocks I. DOI: 10.1107/S1600536808039998/fj2168Isup2.hkl

Additional supplementary materials:  crystallographic information; 3D view; checkCIF report

## Figures and Tables

**Table 1:** Hydrogen-bond geometry (Å, °)

*D*—H⋯*A*	*D*—H	H⋯*A*	*D*⋯*A*	*D*—H⋯*A*
N1—H1⋯O2^i^	0.86	1.95	2.812 (2)	179

Symmetry code: (i) 
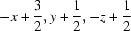
.

**Fig. 1.**
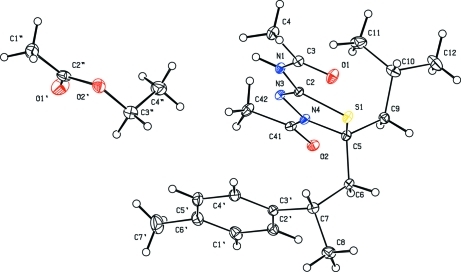
: Molecular structure shown with the atom-labelling scheme. Thermal ellipsoids are drawn at the 30% probability displacement. H atoms are represented as small spheres of arbitrary radius.

**Fig. 2.**
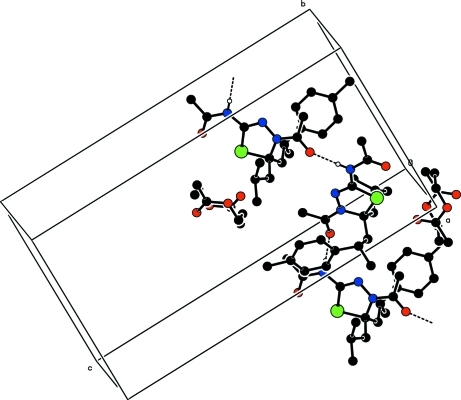
: The crystal packing showing the molecules connected by N–H···O hydrogen bondings (dashed lines). H atoms not involved in hydrogen bonding have been omitted.

## supplementary crystallographic information

### Comment

1,3,4-Thiadiazole derivatives (Sakthivel *et al.*, 2008) are associated with diverse activities: fungicidal (Abdou *et al.*, 1991), pesticidal (Wang *et al.*, 1999, 2004) and bactericidal (Tehranchian *et al.*, 2005) properties.

As part of our ongoing valorization of Cedrus species native to the Medium-Atlas Mountains of Morocco, we have investigated the crystal structure of semi-synthetic terpenoid derivatives obtained through chemical modifications of 1-(4-methylcyclohex-3-enyl) ethanone (I). The latter is isolated from Cedrus Atlantica essential oil. The aromatization of (I) followed by condensation with thiosemicarbazide (Beatriz *et al.*, 2002; Mohammed *et al.*, 2008) ending with treatment by acetic anhydride in the presence of pyridine yielded the compound diastereoisomers in high stereoselectivity.

The title compound molecular structure is shown in figure 1. The solvant molecule is located on the inversion site and disordered. The thiadiazole ring is almost planar with a maximum deviation from the mean plane of 0.015 (2)Å for the C atoms connected to the isobutyl group and a puckering amplitude of 0.026 (2)Å (Cremer & Pople, 1975). Hydrogen bonds are listed in table 1. Investigation of the crystal packing reveals an intermolecular N1—H1···O2 hydrogen bonding generating parallel chains to *b* axis as shown in figure 2.

### Experimental

A solution of 1-(4-methylcyclohex-3-enyl) ethanone (1 g, 4.5 mmol) and Pd/C (10%) were heated at 150°C during 12 h. The product obtained was treated with equimolecular quantity of thiosemicarbazide and several drops of HCl (cc) were added. The reactional mixture was heated at reflux in ethanol for 5 h and then evaporated under reduced pressure and the residue obtained was purified on silica gel column using hexane-ethyl acetate (95:5) as an eluent. 0.25 mmol of the thiosemicarbazone obtained was dissolved in 2 ml of pyridine and 2 ml of acetic anhydride. The mixture was heated on a water bath during 1 h. The resulting residue was concentrated *in vacuo* and chromatographied on silica gel column with hexane-ethyl acetate (90:10) as an eluent. Suitable crystals were obtained by evaporation of ethyl acetate solution at 277 K.

### Refinement

The H atoms linked to the C and N atoms were fixed geometrically and treated as riding with C—H = 0.93 Å (aromatic), 0.96Å (methyl), 0.97 Å (methylene), 0.98Å (methine) and N—H= 0.86 Å with *U*_iso_(H) =1.2*U*_eq_ (aromatic, methylene, methine and NH) or *U*_iso_(H) = 1.5*U*_eq_(methyl).

### Crystal data

**Table:**

C_20_H_29_N_3_O_2_S·0.5C_4_H_8_O_2_	*F*(000) = 904
*M**_r_* = 419.57	*D*_x_ = 1.208 Mg m^−^^3^
Monoclinic, *P*2_1_/*n*	Mo *K*α radiation, λ = 0.71073 Å
Hall symbol: -P 2yn	Cell parameters from 13322 reflections
*a* = 7.8713 (3) Å	θ = 2.6–31.5°
*b* = 12.7587 (5) Å	µ = 0.17 mm^−^^1^
*c* = 22.9688 (9) Å	*T* = 295 K
β = 90.937 (2)°	Prism, colourless
*V* = 2306.39 (16) Å^3^	0.5 × 0.4 × 0.3 mm
*Z* = 4	

### Data collection

**Table:**

Bruker X8 APEX CCD area-detector diffractometer	6688 reflections with *I* > 2σ(*I*)
Radiation source: fine-focus sealed tube	*R*_int_ = 0.037
graphite	θ_max_ = 32.0°, θ_min_ = 1.8°
φ and ω scans	*h* = −11→11
27541 measured reflections	*k* = −16→18
7243 independent reflections	*l* = −33→30

### Refinement

**Table:**

Refinement on *F*^2^	Primary atom site location: structure-invariant direct methods
Least-squares matrix: full	Secondary atom site location: difference Fourier map
*R*[*F*^2^ > 2σ(*F*^2^)] = 0.065	Hydrogen site location: inferred from neighbouring sites
*wR*(*F*^2^) = 0.143	H-atom parameters constrained
*S* = 1.23	*w* = 1/[σ^2^(*F*_o_^2^) + (0.0435*P*)^2^ + 1.5861*P*] where *P* = (*F*_o_^2^ + 2*F*_c_^2^)/3
7243 reflections	(Δ/σ)_max_ = 0.006
298 parameters	Δρ_max_ = 0.48 e Å^−^^3^
0 restraints	Δρ_min_ = −0.35 e Å^−^^3^

### Special details

**Table:**

Geometry. All e.s.d.'s (except the e.s.d. in the dihedral angle between two l.s. planes) are estimated using the full covariance matrix. The cell e.s.d.'s are taken into account individually in the estimation of e.s.d.'s in distances, angles and torsion angles; correlations between e.s.d.'s in cell parameters are only used when they are defined by crystal symmetry. An approximate (isotropic) treatment of cell e.s.d.'s is used for estimating e.s.d.'s involving l.s. planes.
Refinement. Refinement of *F*^2^ against ALL reflections. The weighted *R*-factor *wR* and goodness of fit *S* are based on *F*^2^, conventional *R*-factors *R* are based on *F*, with *F* set to zero for negative *F*^2^. The threshold expression of *F*^2^ > σ(*F*^2^) is used only for calculating *R*-factors(gt) *etc*. and is not relevant to the choice of reflections for refinement. *R*-factors based on *F*^2^ are statistically about twice as large as those based on *F*, and *R*- factors based on ALL data will be even larger.

### Fractional atomic coordinates and isotropic or equivalent isotropic displacement parameters (Å^2^)

**Table:**

	*x*	*y*	*z*	*U*_iso_*/*U*_eq_	Occ. (<1)
C1'	0.5624 (2)	0.56734 (16)	0.09954 (8)	0.0284 (4)	
H1'	0.6130	0.5214	0.0736	0.034*	
C2	0.6060 (2)	0.80786 (11)	0.33245 (7)	0.0160 (3)	
C2'	0.5345 (2)	0.53523 (13)	0.15666 (8)	0.0231 (3)	
H2'	0.5671	0.4681	0.1680	0.028*	
C3	0.4619 (2)	0.92243 (12)	0.40282 (7)	0.0189 (3)	
C3'	0.4581 (2)	0.60205 (12)	0.19769 (7)	0.0198 (3)	
C4'	0.4129 (2)	0.70239 (13)	0.17921 (8)	0.0261 (4)	
H4'	0.3636	0.7488	0.2052	0.031*	
C4	0.4186 (2)	1.03550 (13)	0.41486 (8)	0.0247 (3)	
H41	0.3953	1.0710	0.3788	0.037*	
H42	0.5127	1.0687	0.4345	0.037*	
H43	0.3202	1.0387	0.4389	0.037*	
C5	0.6899 (2)	0.61643 (11)	0.31921 (7)	0.0163 (3)	
C5'	0.4414 (3)	0.73375 (16)	0.12186 (9)	0.0334 (4)	
H5'	0.4100	0.8011	0.1105	0.040*	
C6'	0.5158 (3)	0.66697 (17)	0.08093 (9)	0.0316 (4)	
C6	0.5755 (2)	0.53023 (11)	0.29360 (7)	0.0168 (3)	
H61	0.6439	0.4869	0.2684	0.020*	
H62	0.5382	0.4862	0.3254	0.020*	
C7'	0.5422 (3)	0.6998 (2)	0.01806 (10)	0.0496 (6)	
H71'	0.4499	0.6741	−0.0058	0.074*	
H72'	0.6472	0.6711	0.0046	0.074*	
H73'	0.5461	0.7749	0.0157	0.074*	
C7	0.4196 (2)	0.56528 (12)	0.25922 (7)	0.0199 (3)	
H7	0.3686	0.6241	0.2801	0.024*	
C8	0.2898 (2)	0.47506 (15)	0.25783 (9)	0.0280 (4)	
H81	0.2646	0.4544	0.2969	0.042*	
H82	0.3363	0.4165	0.2372	0.042*	
H83	0.1874	0.4980	0.2384	0.042*	
C9	0.8420 (2)	0.56432 (12)	0.34917 (7)	0.0203 (3)	
H91	0.7995	0.5074	0.3728	0.024*	
H92	0.9113	0.5332	0.3192	0.024*	
C10	0.9575 (2)	0.63119 (15)	0.38756 (8)	0.0260 (4)	
H10	0.8883	0.6640	0.4176	0.031*	
C11	1.0522 (2)	0.71819 (16)	0.35485 (11)	0.0358 (5)	
H111	0.9721	0.7695	0.3410	0.054*	
H112	1.1105	0.6883	0.3224	0.054*	
H113	1.1330	0.7510	0.3807	0.054*	
C12	1.0842 (3)	0.55835 (18)	0.41726 (9)	0.0389 (5)	
H123	1.0243	0.5062	0.4390	0.058*	
H121	1.1563	0.5980	0.4432	0.058*	
H122	1.1522	0.5247	0.3884	0.058*	
C41	0.8340 (2)	0.67172 (12)	0.22639 (7)	0.0182 (3)	
C42	0.8709 (3)	0.75771 (13)	0.18334 (8)	0.0259 (4)	
H423	0.7761	0.8050	0.1811	0.039*	
H421	0.8894	0.7276	0.1457	0.039*	
H422	0.9707	0.7953	0.1958	0.039*	
O1	0.42441 (18)	0.85144 (10)	0.43626 (6)	0.0281 (3)	
O2	0.88630 (16)	0.58120 (9)	0.21879 (5)	0.0211 (2)	
N1	0.54896 (18)	0.90516 (10)	0.35115 (6)	0.0174 (3)	
H1	0.5686	0.9585	0.3294	0.021*	
N3	0.69220 (17)	0.80070 (9)	0.28451 (6)	0.0169 (3)	
N4	0.74060 (18)	0.69617 (9)	0.27477 (6)	0.0167 (3)	
S1	0.56524 (5)	0.69427 (3)	0.372883 (17)	0.01838 (10)	
C1"	0.4182 (8)	0.3287 (4)	0.4750 (2)	0.0377 (11)	0.50
H13"	0.4099	0.3038	0.4357	0.057*	0.50
H12"	0.3115	0.3184	0.4939	0.057*	0.50
H11"	0.5055	0.2906	0.4956	0.057*	0.50
C2"	0.4610 (5)	0.4425 (3)	0.47503 (16)	0.0282 (7)	0.50
C3"	0.4243 (5)	0.6083 (3)	0.52319 (18)	0.0325 (8)	0.50
H32"	0.3404	0.6396	0.5483	0.039*	0.50
H31"	0.4071	0.6367	0.4844	0.039*	0.50
C4"	0.5952 (7)	0.6366 (4)	0.5446 (3)	0.0398 (11)	0.50
H42"	0.6142	0.6066	0.5825	0.060*	0.50
H43"	0.6046	0.7115	0.5470	0.060*	0.50
H41"	0.6784	0.6101	0.5182	0.060*	0.50
O1'	0.5439 (5)	0.4867 (3)	0.43748 (15)	0.0456 (8)	0.50
O2'	0.3987 (4)	0.4949 (2)	0.52147 (12)	0.0326 (6)	0.50

### Atomic displacement parameters (Å^2^)

**Table:**

	*U*^11^	*U*^22^	*U*^33^	*U*^12^	*U*^13^	*U*^23^
C1'	0.0218 (9)	0.0388 (10)	0.0247 (9)	0.0006 (7)	−0.0002 (7)	0.0008 (7)
C2	0.0157 (7)	0.0133 (6)	0.0190 (7)	−0.0004 (5)	0.0000 (5)	0.0009 (5)
C2'	0.0214 (8)	0.0239 (7)	0.0239 (8)	0.0030 (6)	−0.0013 (6)	0.0002 (6)
C3	0.0168 (7)	0.0191 (7)	0.0209 (7)	0.0015 (5)	0.0023 (6)	−0.0008 (5)
C3'	0.0163 (7)	0.0189 (7)	0.0241 (8)	0.0009 (5)	−0.0036 (6)	0.0012 (6)
C4'	0.0244 (9)	0.0213 (7)	0.0324 (9)	0.0032 (6)	−0.0063 (7)	0.0017 (6)
C4	0.0287 (9)	0.0182 (7)	0.0274 (9)	0.0056 (6)	0.0075 (7)	−0.0016 (6)
C5	0.0182 (7)	0.0141 (6)	0.0167 (7)	0.0015 (5)	0.0033 (5)	0.0001 (5)
C5'	0.0314 (10)	0.0288 (9)	0.0396 (11)	−0.0015 (7)	−0.0097 (8)	0.0128 (8)
C6'	0.0237 (9)	0.0410 (10)	0.0300 (10)	−0.0049 (8)	−0.0027 (7)	0.0118 (8)
C6	0.0168 (7)	0.0140 (6)	0.0197 (7)	0.0003 (5)	0.0030 (6)	0.0012 (5)
C7'	0.0455 (14)	0.0672 (17)	0.0360 (12)	−0.0024 (12)	0.0003 (10)	0.0221 (11)
C7	0.0163 (7)	0.0200 (7)	0.0236 (8)	0.0021 (6)	0.0019 (6)	−0.0017 (6)
C8	0.0194 (8)	0.0341 (9)	0.0304 (9)	−0.0049 (7)	0.0026 (7)	0.0002 (7)
C9	0.0213 (8)	0.0194 (7)	0.0201 (7)	0.0027 (6)	−0.0006 (6)	−0.0008 (5)
C10	0.0198 (8)	0.0319 (9)	0.0263 (9)	0.0054 (7)	−0.0023 (7)	−0.0096 (7)
C11	0.0186 (9)	0.0334 (9)	0.0552 (13)	−0.0012 (7)	−0.0059 (9)	−0.0046 (9)
C12	0.0381 (12)	0.0469 (12)	0.0312 (10)	0.0131 (9)	−0.0140 (9)	−0.0090 (9)
C41	0.0189 (7)	0.0165 (6)	0.0192 (7)	−0.0032 (5)	0.0020 (6)	−0.0025 (5)
C42	0.0341 (10)	0.0189 (7)	0.0252 (8)	−0.0031 (6)	0.0116 (7)	−0.0002 (6)
O1	0.0367 (8)	0.0199 (5)	0.0280 (7)	0.0030 (5)	0.0130 (6)	0.0025 (5)
O2	0.0224 (6)	0.0179 (5)	0.0231 (6)	0.0007 (4)	0.0039 (5)	−0.0040 (4)
N1	0.0207 (7)	0.0137 (5)	0.0179 (6)	0.0020 (5)	0.0031 (5)	0.0003 (4)
N3	0.0182 (6)	0.0126 (5)	0.0200 (6)	−0.0002 (5)	0.0020 (5)	−0.0003 (4)
N4	0.0202 (6)	0.0126 (5)	0.0175 (6)	−0.0004 (5)	0.0037 (5)	−0.0007 (4)
S1	0.0229 (2)	0.01389 (16)	0.01860 (18)	0.00144 (13)	0.00611 (14)	0.00131 (12)
C1"	0.057 (3)	0.029 (2)	0.027 (2)	−0.001 (2)	0.001 (2)	0.0012 (17)
C2"	0.0284 (19)	0.0352 (18)	0.0210 (17)	0.0066 (15)	−0.0007 (14)	0.0079 (14)
C3"	0.033 (2)	0.0322 (18)	0.032 (2)	0.0021 (16)	−0.0019 (16)	0.0023 (15)
C4"	0.031 (2)	0.039 (3)	0.048 (3)	0.002 (2)	−0.006 (2)	0.000 (2)
O1'	0.063 (2)	0.0384 (16)	0.0367 (18)	0.0035 (15)	0.0194 (16)	0.0102 (13)
O2'	0.0443 (17)	0.0310 (13)	0.0225 (13)	−0.0037 (12)	−0.0010 (12)	0.0033 (10)

### Geometric parameters (Å, °)

**Table:**

C1'—C6'	1.389 (3)	C8—H83	0.9600
C1'—C2'	1.395 (3)	C9—C10	1.519 (2)
C1'—H1'	0.9300	C9—H91	0.9700
C2—N3	1.306 (2)	C9—H92	0.9700
C2—N1	1.3904 (19)	C10—C12	1.517 (3)
C2—S1	1.7537 (15)	C10—C11	1.540 (3)
C2'—C3'	1.413 (2)	C10—H10	0.9800
C2'—H2'	0.9300	C11—H111	0.9600
C3—O1	1.227 (2)	C11—H112	0.9600
C3—N1	1.398 (2)	C11—H113	0.9600
C3—C4	1.509 (2)	C12—H123	0.9600
C3'—C4'	1.393 (2)	C12—H121	0.9600
C3'—C7	1.524 (2)	C12—H122	0.9600
C4'—C5'	1.398 (3)	C41—O2	1.2394 (18)
C4'—H4'	0.9300	C41—N4	1.378 (2)
C4—H41	0.9600	C41—C42	1.508 (2)
C4—H42	0.9600	C42—H423	0.9600
C4—H43	0.9600	C42—H421	0.9600
C5—N4	1.4998 (19)	C42—H422	0.9600
C5—C9	1.524 (2)	N1—H1	0.8600
C5—C6	1.533 (2)	N3—N4	1.4058 (17)
C5—S1	1.8732 (15)	C1"—C2"	1.490 (6)
C5'—C6'	1.404 (3)	C1"—H13"	0.9600
C5'—H5'	0.9300	C1"—H12"	0.9600
C6'—C7'	1.521 (3)	C1"—H11"	0.9600
C6—C7	1.516 (2)	C2"—O1'	1.227 (5)
C6—H61	0.9700	C2"—O2'	1.358 (5)
C6—H62	0.9700	C3"—O2'	1.461 (5)
C7'—H71'	0.9600	C3"—C4"	1.470 (7)
C7'—H72'	0.9600	C3"—H32"	0.9700
C7'—H73'	0.9600	C3"—H31"	0.9700
C7—C8	1.539 (2)	C4"—H42"	0.9600
C7—H7	0.9800	C4"—H43"	0.9600
C8—H81	0.9600	C4"—H41"	0.9600
C8—H82	0.9600		
			
C6'—C1'—C2'	120.88 (18)	H81—C8—H82	109.5
C6'—C1'—H1'	119.6	C7—C8—H83	109.5
C2'—C1'—H1'	119.6	H81—C8—H83	109.5
N3—C2—N1	119.84 (13)	H82—C8—H83	109.5
N3—C2—S1	119.38 (11)	C10—C9—C5	118.40 (13)
N1—C2—S1	120.77 (12)	C10—C9—H91	107.7
C1'—C2'—C3'	121.70 (16)	C5—C9—H91	107.7
C1'—C2'—H2'	119.2	C10—C9—H92	107.7
C3'—C2'—H2'	119.2	C5—C9—H92	107.7
O1—C3—N1	122.80 (14)	H91—C9—H92	107.1
O1—C3—C4	122.25 (15)	C12—C10—C9	107.44 (15)
N1—C3—C4	114.95 (14)	C12—C10—C11	109.96 (17)
C4'—C3'—C2'	117.43 (16)	C9—C10—C11	114.31 (16)
C4'—C3'—C7	120.79 (16)	C12—C10—H10	108.3
C2'—C3'—C7	121.74 (14)	C9—C10—H10	108.3
C3'—C4'—C5'	120.40 (18)	C11—C10—H10	108.3
C3'—C4'—H4'	119.8	C10—C11—H111	109.5
C5'—C4'—H4'	119.8	C10—C11—H112	109.5
C3—C4—H41	109.5	H111—C11—H112	109.5
C3—C4—H42	109.5	C10—C11—H113	109.5
H41—C4—H42	109.5	H111—C11—H113	109.5
C3—C4—H43	109.5	H112—C11—H113	109.5
H41—C4—H43	109.5	C10—C12—H123	109.5
H42—C4—H43	109.5	C10—C12—H121	109.5
N4—C5—C9	112.80 (13)	H123—C12—H121	109.5
N4—C5—C6	112.75 (12)	C10—C12—H122	109.5
C9—C5—C6	108.21 (12)	H123—C12—H122	109.5
N4—C5—S1	103.67 (9)	H121—C12—H122	109.5
C9—C5—S1	110.51 (11)	O2—C41—N4	120.49 (14)
C6—C5—S1	108.79 (11)	O2—C41—C42	121.15 (15)
C4'—C5'—C6'	122.16 (17)	N4—C41—C42	118.36 (13)
C4'—C5'—H5'	118.9	C41—C42—H423	109.5
C6'—C5'—H5'	118.9	C41—C42—H421	109.5
C1'—C6'—C5'	117.43 (18)	H423—C42—H421	109.5
C1'—C6'—C7'	120.3 (2)	C41—C42—H422	109.5
C5'—C6'—C7'	122.3 (2)	H423—C42—H422	109.5
C7—C6—C5	116.99 (12)	H421—C42—H422	109.5
C7—C6—H61	108.1	C2—N1—C3	124.63 (13)
C5—C6—H61	108.1	C2—N1—H1	117.7
C7—C6—H62	108.1	C3—N1—H1	117.7
C5—C6—H62	108.1	C2—N3—N4	110.27 (12)
H61—C6—H62	107.3	C41—N4—N3	119.50 (12)
C6'—C7'—H71'	109.5	C41—N4—C5	123.13 (12)
C6'—C7'—H72'	109.5	N3—N4—C5	117.36 (12)
H71'—C7'—H72'	109.5	C2—S1—C5	89.27 (7)
C6'—C7'—H73'	109.5	O1'—C2"—O2'	121.9 (4)
H71'—C7'—H73'	109.5	O1'—C2"—C1"	124.8 (4)
H72'—C7'—H73'	109.5	O2'—C2"—C1"	113.3 (4)
C6—C7—C3'	113.72 (14)	O2'—C3"—C4"	112.2 (4)
C6—C7—C8	108.77 (13)	O2'—C3"—H32"	109.2
C3'—C7—C8	110.65 (14)	C4"—C3"—H32"	109.2
C6—C7—H7	107.8	O2'—C3"—H31"	109.2
C3'—C7—H7	107.8	C4"—C3"—H31"	109.2
C8—C7—H7	107.8	H32"—C3"—H31"	107.9
C7—C8—H81	109.5	C2"—O2'—C3"	117.3 (3)
C7—C8—H82	109.5		

### Hydrogen-bond geometry (Å, °)

**Table:**

*D*—H···*A*	*D*—H	H···*A*	*D*···*A*	*D*—H···*A*
N1—H1···O2^i^	0.86	1.95	2.812 (2)	179

Symmetry codes: (i) −*x*+3/2, *y*+1/2, −*z*+1/2.
